# Understanding the CO Oxidation on Pt Nanoparticles Supported on MOFs by *Operando* XPS

**DOI:** 10.1002/cctc.201801067

**Published:** 2018-08-13

**Authors:** Reza Vakili, Emma K. Gibson, Sarayute Chansai, Shaojun Xu, Nadeen Al‐Janabi, Peter P. Wells, Christopher Hardacre, Alex Walton, Xiaolei Fan

**Affiliations:** ^1^ School of Chemical Engineering and Analytical Science The University of Manchester Oxford Road Manchester M13 9PL UK; ^2^ School of Chemistry University of Glasgow University Avenue Glasgow G12 8QQ UK; ^3^ UK Catalysis Hub Research Complex at Harwell Rutherford Appleton Laboratory Harwell Oxon Didcot OX11 0FA UK; ^4^ Chemistry University of Southampton Highfield Southampton SO17 1BJ UK; ^5^ School of Chemistry The University of Manchester Oxford Road Manchester M13 9PL UK; ^6^ Institution Photon Science Institute The University of Manchester Oxford Road Manchester M13 9PL UK

**Keywords:** Metal-Organic Frameworks (MOFs), Pt Catalysts, *Operando* Near Ambient Pressure XPS (NAP-XPS), CO Oxidation, Confinement Effect

## Abstract

Metal‐organic frameworks (MOFs) are playing a key role in developing the next generation of heterogeneous catalysts. In this work, near ambient pressure X‐ray photoelectron spectroscopy (NAP‐XPS) is applied to study in *operando* the CO oxidation on Pt@MOFs (UiO‐67) and Pt@ZrO_2_ catalysts, revealing the same Pt surface dynamics under the stoichiometric CO/O_2_ ambient at 3 mbar. Upon the ignition at *ca*. 200 °C, the signature Pt binding energy (BE) shift towards the lower BE (from 71.8 to 71.2 eV) is observed for all catalysts, confirming metallic Pt nanoparticles (NPs) as the active phase. Additionally, the plug‐flow light‐off experiments show the superior activity of the Pt@MOFs catalyst in CO oxidation than the control Pt@ZrO_2_ catalyst with *ca*. 28 % drop in the *T*_50%_ light‐off temperature, as well as high stability, due to their sintering‐resistance feature. These results provide evidence that the uniqueness of MOFs as the catalyst supports lies in the structural confinement effect.

MOFs are promising porous solids for heterogeneous catalysis. The hydrothermal stability of metal organic frameworks (MOFs) can be improved significantly using metals with high oxidation states such as Zr^4+^ and Al^3+^ to reinforce the coordinative bonds,^1^ allowing the development of practical MOF‐based catalysts. In this respect, Zr‐based UiO (UiO for Universitetet i Oslo) MOFs^2^ have been widely used as hosts for dispersing metal nanoparticles (MNPs) for heterogeneous catalysis^3^ and have demonstrated remarkable stability (up to 450 °C for UiO‐67) under thermal activation.^4^

Broadly, the major approaches to stabilise MNPs within UiO MOFs include (i) post‐synthetic deposition and (ii) direct incorporation methods. Post‐synthetic routes (*e*. *g*. wetness impregnation) are attractive due to simplicity and easy scale‐up.^5^ However, the diffusion resistance between the external and internal surfaces3a may result in the preferential MNPs deposition on the external surface of MOFs. Direct incorporation provides an elegant way of integrating MNPs or MNPs precursors within the framework during the synthesis of MOFs,4a theoretically enabling the uniform distribution of MNPs throughout the resulting catalysts. MNPs incorporated in UiO MOFs catalysts have been tested for various reactions,3a–3d,3f,^6^ showing generally good catalytic performance. For example, fine Pd NPs of 1.5 nm supported on UiO‐67 showed high selectivity (>99 %) in C=C bond hydrogenation,3a thanks to the confinement effect of MOFs, limiting the size of MNPs by their well‐defined pore network.

Although few efforts were made to gain insight into the property (*e*. *g*. size and shape), chemical state and local environment of MNPs in MOFs, most of the studies have focused on the pre and post reaction characterisation of catalysts,^7^ providing snapshots only of their properties. Moreover, experimental observation of the working state of MNPs in MOFs in comparison with conventional supports such as metal oxides is still largely lacking.^8^ Herein, we report the first comparative study of platinum (Pt) NPs supported on UiO‐67 and zirconia (ZrO_2_) using *operando* near‐ambient pressure X‐ray photoelectron spectroscopy (NAP‐XPS) with simultaneous mass spectrometry to elucidate the role of MOFs as catalyst supports.

We prepared Pt NPs (*ca*. 2 wt.%) supported on UiO‐67 using the wetness impregnation (WI‐PtNPs@UiO‐67) and linker design (LD‐PtNPs@UiO‐67) methods,3a,3b with Pt NPs supported on ZrO_2_ as the control catalyst (PtNPs@ZrO_2_, Supplementary Information (SI)). The catalytic activity was probed using CO oxidation, in which LD‐PtNPs@UiO‐67 catalyst showed enhanced CO turnover frequency (TOF) relative to PtNPs@ZrO_2_ by *ca*. 12 % and are surprisingly stable under CO oxidation conditions. *Operando* XPS measurements revealed the same underlying surface chemistry for the catalysts, clearly showing the active metallic Pt NPs under catalytic conditions. The higher catalytic activity for the MOF samples must, therefore, arise from the improved dispersion of the Pt and retained NP size due to the structural confinement effect.

The as‐synthesized catalysts were reduced *in situ* before the *operando* XPS analysis, as in Figures 1a and 1b show mixed Pt oxidation states in the as‐synthesized MOF catalysts. Pt 4f core level shows clearly the presence of both two chemical species at 71.2±0.1 eV and a higher binding energy (BE) peak at 72.6±0.1 eV. The higher peak is consistent with PtO (or other Pt(II) species).^9^ The lower binding energy peak is slightly above the reference value for bulk Pt metal of 71.0 eV,^10^ which we assign to Pt(0) NPs. Small Pt NPs are known to exhibit BEs higher than bulk Pt due to initial and final state effects arising from their very small spatial extent.^10^, ^11^

WI‐Pt@UiO‐67 can be reduced upon heating under vacuum due to the ketone photosensitization mechanism,^12^ since acetone is the main product from the thermal decomposition of metal acetylacetonates.^13^ By heating the catalyst *in situ* to 200 °C under vacuum, the higher BE components disappear, leaving only the metallic Pt 4f doublet (Figure 1a), confirming the formation of Pt NPs in the WI‐PtNPs@UiO‐67 catalyst. In NAP‐XPS, the complete reduction of LD‐Pt@UiO‐67 (denoted as LD‐PtNPs@UiO‐67) is achieved at 250 °C and 1 mbar H_2_ (Figure 1b). Transmission electron microscopy (TEM) analysis (Figures 1c, 1d and S2a) shows that the reduced catalysts possess dispersed Pt NPs with different sizes in the hosts. The LD method promoted finer Pt NPs of 1.2±0.4 nm than the WI method, by which Pt NPs with average sizes of 2.5±0.7 nm in UiO‐67 and 1.9±1.2 on ZrO_2_ were produced (as shown in the insets of Figures 1c, 1d and S2a).

**Figure 1:**
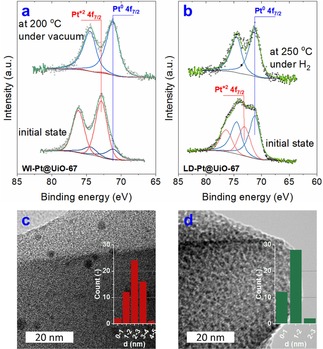
Pt 4f spectra of (a) WI‐Pt@UiO‐67 at different temperatures under vacuum; and (b) LD‐Pt@UiO‐67 at different temperatures and 1 mbar H_2_; TEM micrographs for (c) WI‐PtNPs@UiO‐67 and (d) LD‐PtNPs@UiO‐67 catalysts (Insets: Pt NPs size distribution histograms).

XPS is a surface sensitive technique due to the short inelastic mean free path (IMFP) of the emitted photoelectrons. In Pt metal, there is a short IMFP of 16 Å for Pt 4f photoelectrons.^14^ This gives an information depth of *ca*. 5 nm into the particles. Given that the average size of all Pt NPs in this report is smaller than this, we assume that the Pt 4f spectra recorded are sampling the entire nanoparticle (averaged surface/bulk) and not just the surface region.

To understand the chemical state of Pt NPs in UiO‐67 during catalytic turnover, we analysed the Pt 4f region using *operando* XPS in the temperature‐programmed measurements (100–260 °C, SI) with reference to PtNPs@ZrO_2_ (2 wt.%) catalyst. CO conversion during NAP‐XPS experiments with the stoichiometric mixture (Figure 2a) shows that WI‐PtNPs@UiO‐67 has a better CO turnover frequency (TOF, 0.066 s^−1^ at 260 °C) than the other two (*ca*. 0.055 s^−1^). Pt 4f peaks only show one chemical species present at all temperatures (Figures 2b and S4), but the core level BE shifts change as a function of reaction temperature, *i*. *e*. Pt 4f peak from 71.8 eV at T<200 °C to 71.2 eV at T>200 °C (Figures 2b and 2c). This BE shift is the same for all catalysts (Figure 2b) and corresponds with the onset of CO conversion for them (Figure 2a). The peak positions of the Zr 3d_5/2_ peak for MOFs based catalysts (Figure 2d) show no corresponding shift, confirming that the shift in the Pt 4f peak reflects a change in chemical state of the Pt NPs rather than relating to any charging phenomena.

**Figure 2:**
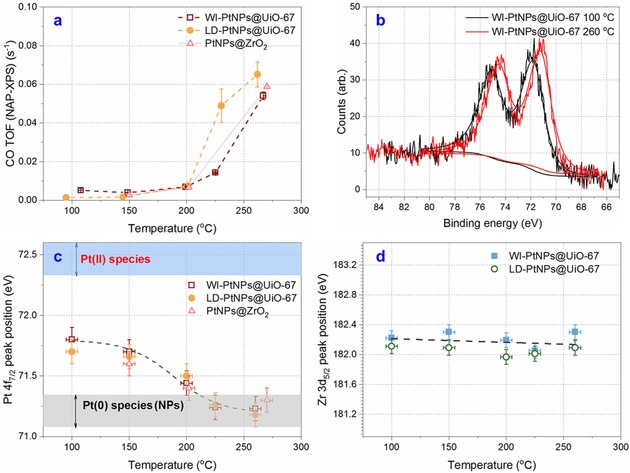
*Operando* XPS temperature programmed CO oxidation over PtNPs@UiO‐67 catalysts (CO/O_2_ ratio=2, total pressure=3 mbar). (a) CO conversion for each temperature measured for both WI and LD catalysts; (b) Example Pt 4f spectra from the WI catalysts before and during conversion, showing the BE shift; (c) Measured Pt 4f_7/2_ peak position as a function of temperature; (d) BE of the Zr 3d peak as a function of temperature, showing no shift.

The findings from the *operando* NAP‐XPS study suggest that, in the active regime, the metallic, adsorbate‐free surface of Pt NPs is the active phase for CO oxidation and adsorbed CO has a very short residence time before oxidation. This result is in good agreement with the findings on the Pt NPs/TiO_2_(110) catalyst (at 1 mbar and CO/O_2_=1 : 4)^15^ and the model Pt(111) crystals (at 1 mbar stoichiometric CO/O_2_),^16^ but contradicting to the conjecture of Pt oxides as reactive phases in the model sPt (111) and Pt(110) catalysts.^17^ NAP‐XPS studies of catalytic CO oxidation were mainly conducted using model catalysts,^18^ because natural Pt NPs have various facets, increasing the complexity of understanding molecule binding on these facets. Considering small Pt NPs (*ca*. 2 nm), one can assume that Pt(111) facets dominate in these Pt NPs.^15^

BE shift observed for Pt 4f_7/2_ (Figure 2b) does not reflect the reduction of Pt species from Pt(II) to Pt(0) bulk. BEs at <200 °C are too high for metallic Pt (71.2 eV^19^), as well as too low for Pt(II) species which have BEs starting at 72.4 eV.^9^ BEs at >200 °C are consistent with metallic Pt NPs.^19^ Previously, organic ligands functionalized Pt NPs (<2 nm) were reported to have BE intermediates between Pt(0) and Pt(II) due to the charge transfer between the particle and surface‐bound ligands.^20^ We propose that a similar effect is occurring here: at low temperatures the surface of Pt NPs is covered with CO adsorbate molecules, causing this charge transfer effect and blocking the surface such that catalytic turnover cannot take place. As this higher BE state of 71.8 eV was not observed after the *in situ* reduction of the catalysts and only once the CO/O_2_ mixture was introduced, we attribute it to the surface saturation of Pt NPs with adsorbed CO. At higher temperatures, this CO is desorbed and a bare Pt surface achieved, causing the down‐shift in Pt 4f_7/2_ and the onset of CO conversion. The observed BE shift cannot be related to Pt NPs size effects (the BE would also shift if the particle size substantially changes, as discussed earlier) as the post‐mortem TEM shows no significant change in particle size distribution before and after the catalytic testing (by comparing Figures 1c and 1d with Figures 3b and 3c).

The sudden switch in BE, corresponding to the change of surface coverage from adsorbed CO to adsorbed dissociated oxygen and light‐off, was also seen on the model Pd(110) by measuring O 1s region using temperature‐programmed XPS.^21^ However, in this work, the data from region scans of the O 1s and C 1s core levels are not taken into account in the quantitative analysis of the catalyst surfaces due to the presence of much larger amounts of C and O in UiO‐67, leading to the difficulty to resolving the O 1s and C 1s peaks.

In general, the quantitative analysis of Pt 4f data from the *operando* XPS experiment shows that the surface chemistry of the two Pt catalysts supported on UiO‐67 is the same, comparable to that of PtNPs@ZrO_2_. The observed difference in the catalytic activity can be attributed to the particle sizes of Pt NPs (insets in Figures 1c and 1d), as well as the dispersion of active phase in the support (SI), which are different in the three catalysts.

A comparative study of the three catalysts using the light‐off experiments (CO/O_2_=2) was performed in a plug‐flow reactor to assess their catalytic activities in applications (the light‐off curves as in Figure 3a). *T*_50%_ light‐off temperatures (inset of Figure 3a) were measured at 247, 284 and 341 °C for LD‐PtNPs@UiO‐67, WI‐PtNPs@UiO‐67 and PtNPs@ZrO_2_ catalysts, respectively, showing that the LD‐PtNPs@UiO‐67 catalyst has the highest activity for CO oxidation under the plug flow condition. CO conversions from the plug‐flow reactor (*ca*.70 % for the LD‐PtNPs@UiO‐67 at 260 °C) are higher than that measured by the *operando* NAP‐XPS (*ca*. 26 %). This is attributed to the difference in configurations of the two systems (SI and Figure S5). Nevertheless, both experiments confirm the higher catalytic activity of LD‐PtNPs@UiO‐67 over the other two catalysts.

**Figure 3:**
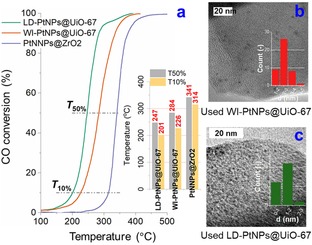
(a) Light‐off curves (CO conversion efficiencies) of CO oxidation over different Pt NPs catalysts (inset: comparison of *T*_50%_ and *T*_10%_). Conditions: heating ramp=6 °C min^−1^, atmospheric pressure, total flowrate=100 ml min^−1^, CO/O_2_=2, balanced using Ar; TEM micrographs for (b) used WI‐PtNPs@UiO‐67 and (c) used LD‐PtNPs@UiO‐67 catalysts (after five cycles of CO oxidation).

The MOF catalysts demonstrate good stability in the reusability tests (Figure 4), showing no deactivation over five cycles of light‐off experiments (under the lean condition, which is relevant to practical applications). Conversely, *T*_50%_ of PtNPs@ZrO_2_ increases by 7 % after five runs. Post reaction TEM examination of the used catalysts (after five cycles) shows that UiO‐67 prevents the sintering of Pt NPs effectively (1.22±0.35 and 2.54±0.62 nm, respectively, similar to that of fresh catalysts), even at 300 °C under oxidising conditions, as shown in Figures 3b and 3c. However, significant Pt NP sintering is found for ZrO_2_, leading to an increase in NP size by one order of magnitude (Figure S3b). Therefore, the use of MOFs as catalyst supports is particularly beneficial over oxide supports thanks to the structural confinement effect limiting the growth of NPs. The intrinsic nature of the active phases (as revealed by the *operando* XPS study) is comparable to relevant model Pt catalysts.^16^

**Figure 4:**
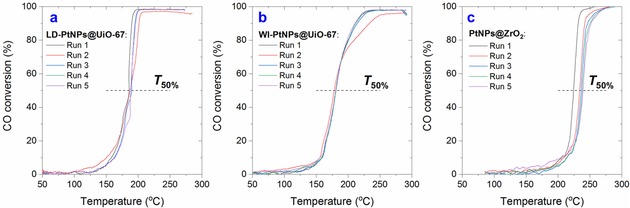
Light‐off curves of CO oxidation over (a) LD‐PtNPs@UiO‐67, (b) WI‐PtNPs@UiO‐67 and (c) PtNPs@ZrO_2_ catalysts. Conditions: heating ramp=6 °C min^−1^, atmospheric pressure, total flowrate=100 ml min^−1^, CO/O_2_=0.2, balanced using Ar.

In conclusion, we have demonstrated the practice of XPS study of CO oxidation over Pt catalysts incorporated in UiO‐67 MOF under CO/O_2_ ambient, showing the feasibility and ability to use NAP‐XPS for understanding MOFs‐based catalysts in *operando*. By examining the working Pt supported on MOF catalysts in CO oxidation against the Pt@ZrO_2_ catalyst, we found the similar underlying surface chemistry of these catalysts. Pt catalyst supported on UiO‐67 demonstrate much better sinter‐resistance and stability, as well as higher activity, than that of Pt@ZrO_2_ in the light‐off experiment under plug‐flow conditions, thanks to the well‐defined porous framework of UiO‐67. These findings give a precise definition of the significance of using stable MOFs as catalyst supports, *i*. *e*. the structural confinement effect which inhibits catalyst sintering.

## Conflict of interest

The authors declare no conflict of interest.

## Supporting information

As a service to our authors and readers, this journal provides supporting information supplied by the authors. Such materials are peer reviewed and may be re‐organized for online delivery, but are not copy‐edited or typeset. Technical support issues arising from supporting information (other than missing files) should be addressed to the authors.

Supplementary
