# Supplementary material for: Understanding the CO Oxidation on Pt Nanoparticles Supported on MOFs by Operando XPS
Source: ChemCatChem. 2018 Aug 13;10(19):4238–42. doi: 10.1002/cctc.201801067 (PMC6470863; doi:10.1002/cctc.201801067)
Supplement: Supplementary file 1 — Supplementary [file CCTC-10-4238-s001.pdf]

Supporting Information

© Copyright Wiley-VCH Verlag GmbH & Co. KGaA, 69451 Weinheim, 2018

Understanding the CO Oxidation on Pt Nanoparticles Supported on MOFs by *Operando* XPS

Reza Vakili, Emma K. Gibson, Sarayute Chansai[+], Shaojun Xu[+], Nadeen Al-Janabi, Peter P. Wells, Christopher Hardacre, Alex Walton,* and Xiaolei Fan*

Author Contributions

X.F. Formal analysis:Lead; Project administration:Lead; Supervision:Lead; Writing – original draft:Equal; Writing – review & editing:Lead
R.V. Investigation:Lead; Methodology:Supporting; Visualization:Supporting; Writing – original draft:Supporting
E.G. Formal analysis:Equal; Visualization:Supporting; Writing – review & editing:Supporting
S.C. Investigation:Equal; Methodology:Equal
S.X. Investigation:Equal; Methodology:Equal
P.W. Investigation:Equal; Methodology:Supporting; Writing – review & editing:Supporting
C.H. Supervision:Supporting; Writing – review & editing:Supporting
A.W. Conceptualization:Supporting; Formal analysis:Equal; Investigation:Supporting; Methodology:Lead; Writing – original draft:Equal; Writing – review & editing:Supporting
N.A.-J. Investigation:Supporting

Understanding the CO Oxidation on Pt Nanoparticles Supported on MOFs by Operando XPS

Supporting information

Catalysts preparation:

The wetness impregnation (WI) method was undertaken using (*i*) platinum(II) acetylacetonate ($Pt(acac)_2$) in acetone to impregnate the pre-synthesised UiO-67[1] and (*ii*) potassium tetrachloroplatinate(II) (K_2PtCl_4) in water to impregnate the the commercial ZrO_2. The linker design (LD) method incorporates Pt species (H_2PtCl_4 as the precursor) by grafting the Pt precursors to 2,2-bipyridine-5,5-dicarboxylic acid (bpydc) linker.[2] The detailed procedures of the catalyst synthesis are described as:

2 wt.% Pt supported on UiO-67 by the WI method (WI-Pt@UiO-67): UiO-67 support was synthesised by a microwave-assisted method proposed in our previous work.[1] To prepare WI-Pt@UiO-67, 4 mg $Pt(acac)_2$ was dissolved in 2 ml acetone, then Pt precursor solution was dropped on 100 mg UiO-67 support stepwise. When the sample was fully moistened with the Pt precursor solution, it was dried in an oven at 50 °C before the next deposition. The WI-Pt@UiO-67 was reduced by thermal treatment at 200 °C under vacuum to give WI-PtNPs@UiO-67 (heating ramp: 3 ºC min^{-1}, vacuum pressure: <20 mbar).

2 wt.% Pt supported on ZrO_2 the WI method (Pt@ZrO_2): K_2PtCl_4 (21.3 mg) was dissolved in water (2 ml) to form the precursor solution, then it was added dropwise on the commercial ZrO_2 (500 mg) and dried to prepare Pt@ZrO_2. Pt@ZrO_2 was reduced at 280 °C under hydrogen (H_2)-argon flow (flowrate: 70 ml min^{-1} (10% H_2), heating ramp: 5 °C min^{-1}) to give PtNPs@ZrO_2

$PtCl_2$(bpydc) linker design: 2,2-Bipyridine-5,5-dicarboxylic acid (bpydc) linkers were used to incorporate Pt precursors. $PtCl_2$(bpydc) was synthesized by adding bpydc (1.04 mmol, 1 equiv), K_2PtCl_4 (1.21 mmol, 1.2 equiv), and HNO_3 (1 mL, 0.1 M) into a 150 ml round-bottomed flask

containing water (100 ml). The mixture was heated to 100 °C and stirred under reflux for 40 h. After the synthesis, the resulting precipitate was filtered and washed using deionised water and then dried in an oven at 70°C. A $PtCl_2$(bpydc) yield of *ca.* 75% (based on the initial amount of K_2PtCl_4) was achieved, which is close to the yield reported in the literature.[2a]

2 wt.% Pt supported on UiO-67 by the LD (LD-Pt@UiO-67): To a 100 ml Teflon-lined autoclave reactor containing 50 mL N,N-dimethylformamide was added $ZrCl_4$ (1.28 mmol, 1 equiv), 4,4-biphenyl-dicarboxylic acid (bpdc) (1.23 mmol), and benzoic acid (12.8, 10 equiv.). The resulting solution was sonicated for 10 min to dissolve the suspended particles until an opaque white solution was obtained. The reactor was heated at 95 °C (in an oil bath) for 4 days with the lid tightly sealed. After the synthesis, the solution was allowed to cool to room temperature, and the liquid was decanted. The solid particles were exchanged for fresh ethanol several times and then filtered and dried under vacuum at 100 °C. LD-Pt@UiO-67 was reduced at 262 °C under hydrogen-argon flow (flowrate: 70 ml min^{-1} (5% H_2), heating ramp: 5 °C min^{-1}) to give LD-PtNPs@UiO-67.

Materials characterisation:

X-ray diffraction (XRD) of materials was carried out on a Rigaku Miniflex diffractometer using CuKα_1 radiation (λ = 0.15406 nm, 30 kV, 15 mA). The measurement was perforce over a range of 4° < 2θ < 45° in 0.05 step size at a scanning rate of 1° min^{-1}. Scanning electron microscopy (SEM) was undertaken using a FEI Quanta 200 ESEM equipment using a work distance of 8–10 mm and an accelerating voltage of 20 kV. All samples were dispersed in ethanol and dropped onto SEM stubs, followed by the gold coating using an Emitech K550X sputter coater under vacuum (1×10^{-4} mbar). Nitrogen (N_2) sorption on materials at −196.15 °C was carried out using a Micromeritics ASAP 2020 analyser. Prior to the N_2 adsorption, samples (~100 mg) were pretreated by degassing at 200°C under vacuum overnight. The surface area and total pore volume of the materials were calculated based on Brunauer–Emmett–Teller (BET) theory and at a relative pressure P/P^0 of 0.99, respectively. Thermogravimetric analysis (TGA) and was performed by use of a TG analyser

(Beijing Boyuan Science and Technology Development Co., Ltd) from the room temperature to 700 °C in air (flowrate = 0.6 ml min^{-1}) at a heating rate of 5 °C min^{-1}.

The Pt content of the synthesised catalysts was determined by inductively coupled plasma optical emission spectrometry (ICP-OES, Thermo iCAP 6000 SERIES). Samples were digested in the nitric acid solution overnight then solutions were analysed by ICP for the quantitative determination of the Pt contents. It was determined that about 2 wt.% Pt species were present in all catalysts.

***Operando* near ambient pressure XPS (NAP-XPS) study:**

All XPS spectra were recorded with a SPECS NAP-XPS system employing a monochromatic Al Kα source (1486.6 eV). The catalyst powders were dispersed in ethanol and then drop-cast onto a silicon substrate. The drop-casting was repeated until a uniform layer of MOF covered the silicon pieces and no significant Si 2p signal was observable in XPS. The insulating nature of these MOFs presents a challenge to NAP-XPS analysis as conventional charge compensation mechanisms (electron flood sources) are not usable within the high-pressure environment of a NAP-XPS. In vacuum conditions, the samples charged by ~50 eV and substantial differential charging make the spectra unusable. However, it was found by admitting an appropriate amount of gas into the NAP cell and heating the sample, the sample could be fully charge compensated and usable spectra obtained. The spectra were recorded at a pass energy of 30 eV and charge corrected to the main component of the C 1s peak at 284.5 eV for aromatic carbon (the main constituent of the 4,4’-biphenyl-dicarboxylic acid, bpdc, linker in UiO-67). The Zr 3d peak was used as a check to ensure that no differential charging was occurring. Its position was 182.2 ± 0.1 eV and did not significantly change throughout the experiment (see Fig 2d in main text), confirming that the shifts on the Pt 4f were due to real physical/chemical changes and not related to differential charging.

The NAP-XPS was performed during catalyst exposure to a mixture of $CO:O_2$ (CO/O_2 ratio = 2, total pressure = 3 mbar) at room temperature before it was heated in steps to 260 °C, with XPS spectra acquired at each temperature. The C 1s and O 1s core levels were also acquired, but

information about C and O surface species on the Pt particles could not be obtained as the signal was dominated by the C and O in the bpdc linker. The acquisition time for the XPS spectra at each temperature was about 2 h in the temperature-programmed measurements (100–260 °C). XPS measurements were taken at 100 °C, 150 °C, 200 °C, 225 °C and 260 °C, respectively, to probe the chemical state of the Pt catalysts. Online gas analysis of the CO oxidation over the catalysts was monitored using a quadrupole mass spectrometer (QMS) (MKS EasyView 2) in the second differential stage of the NAP hemispherical analyser.

CO oxidation reaction the quartz plug-flow reactor:

The reaction was carried out in a quartz tubular reactor (9.0 mm ID) placed inside a programmable furnace at atmospheric pressure and with a flowrate of 100 ml min^{-1} (20 sccm CO, 10 sccm O_2 and 70 sccm Ar). 50 mg of the pelletised catalysts (*i.e.* WI-PtNPs@UiO-67, LD-PtNPs@UiO-67 or PtNPs@ZrO_2) were packed into the reactor and sandwiched by the quartz wool. Prior to the reaction, the catalyst was treated for 1 h in a reducing environment with the hydrogen-argon (Ar) flow (10 vol.% H_2, the total flowrate of 100 ml min^{-1}, at 280 °C). The reaction temperature was ramped from the room temperature to 380 °C (for UiO MOFs catalysts) and to 580 °C (for PtNPs@ZrO_2) at a heating rate of *ca.* 8 °C min^{-1}. The bed temperature was measured and recorded by a K type thermocouple adjacent to the catalyst bed. After each run, the furnace was turned off automatically to allow the reactor to cool down to the room temperature under Ar (at 100 ml min^{-1}). Mass spectrometry (MS) of the products was measured using an HPR20 QIC Hiden Analytical mass spectrometer. During the experiments, the spectrometer continuously monitored the ion currents at a mass-to-charge ratio (m/e) = 36, 18, 28, 32 and 44, corresponding to signals from Ar, H_2O, CO, O_2 and CO_2, respectively. For cyclic catalyst deactivation tests, the same heating ramp and reactant gases were applied to the bed when the room temperature was achieved after each run.

Turnover frequencies (TOFs) calculation:

Turnover frequencies (TOFs) are calculated as the normalised activity for CO oxidation to the number of estimated available Pt surface atoms on the Pt NPs, as in Eq. (1):

$$\mathrm{TOF} = \frac{R_{\mathrm{Pt}} \times M_{\mathrm{Pt}}}{D_{\mathrm{Pt}}} \tag{1}$$

where M_{Pt} is the molar mass of Pt (195 g mol^{-1}), D_{Pt} is the Pt dispersion of the catalyst (-, estimated using the method below); and R_{Pt} is the Pt mass normalised reaction rate (mol s^{-1} $g_{\mathrm{catal.}}{}^{-1}$), defined as in Eq. (2):

$$R_{\mathrm{Pt}} = \frac{X_{\mathrm{CO}} \times \dot{n}_{\mathrm{CO}}}{m_{\mathrm{Pt}}} \tag{2}$$

where of X_{CO} is the measured CO conversion (-); $\dot{n}_{\mathrm{CO}}$ is the molar flow rate of CO into the system (mol s^{-1}); and m_{Pt} is the Pt mass present in the catalyst bed (g).

Pt dispersion estimation:

Calculation of Pt dispersion (D_{Pt}) for TOF estimation:

Pt has a face-centred cubic (fcc) unit cell with the edge length of 0.392 nm (4 Pt atoms per unit cell). Therefore, Pt fcc unit cell volume is:

$0.392^3 = 0.06$ nm^3

Consider the Pt NPs are spherical. The volumes of the catalysts are:

WI-PtNPs@UiO-67 = $4/3\times\pi\times(1.25)^3 = 8.18$ nm^3

LD-PtNPs@UiO-67 = $4/3\times\pi\times(0.6)^3 = 0.905$ nm^3

PtNPs@ZrO_2 = $4/3\times\pi\times(0.8)^3 = 2.14$ nm^3

Therefore, the approximate numbers of fcc unit cells and Pt atoms in Pt NPs can be estimated as:

WI-PtNPs@UiO-67 = 8.18 nm^3/0.06 nm^3 = 136 unit cells = 544 Pt atoms

LD-PtNPs@UiO-67 = 0.905 nm^3/0.06 nm^3 = 15 unit cells = 60 Pt atoms

PtNPs@ZrO_2 = 2.14 nm^3/0.06 nm^3 = 36 unit cells = 143 Pt atoms

The number of atoms on the sliding surface is determined by multiplying of contact area values by the planar density of platinum atoms on (111) planes, which is 12 atoms/nm^2:[3]

WI-PtNPs@UiO-67 = $4\times\pi\times(1.25)^2\times12$ = 236 Pt atoms

LD-PtNPs@UiO-67 = $4\times\pi\times(0.6)^2\times12$ = 54 Pt atoms

PtNPs@ZrO_2 = $4\times\pi\times(0.8)^2\times12$ = 97 Pt atoms

Assume hemispherical Pt particles sitting on the support after the reduction. The dispersion of Pt can be estimated as:

WI-PtNPs@UiO-67 = 236/2/544 = 22%

LD-PtNPs@UiO-67 = 54/2/60 = 45%

PtNPs@ZrO_2 = 97/2/143 = 34%

It is noteworthy that the complete reduction of the bulk LD-Pt@UiO-67 was measured at 262 °C by temperature programmed reduction (under 10 vol.% H_2 in Helium, He, Figure S1), showing the comparable reducibility of the bulk and surface of LD-Pt@UiO-67. The difference in the reduction temperatures is attributed to the difference in H_2 partial pressure and reactor configuration.[4]

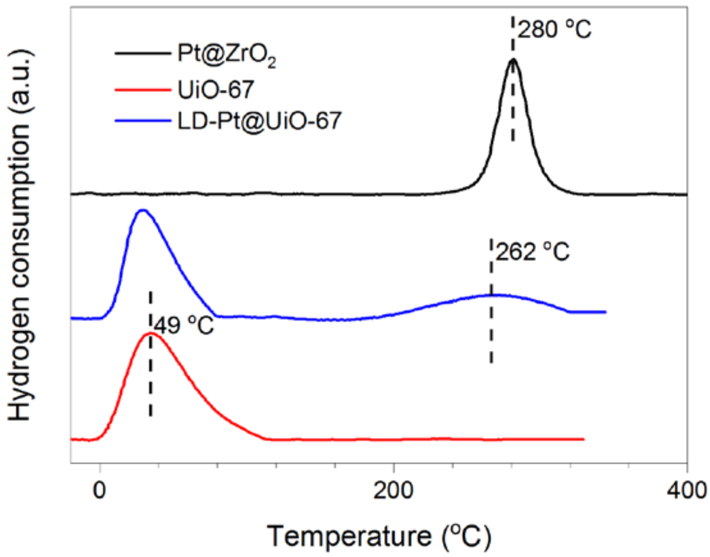

Figure S1. H_2 temperature programmed reduction profiles for UiO-67, LD-Pt@UiO-67 and WI-Pt@ZrO$_2$.

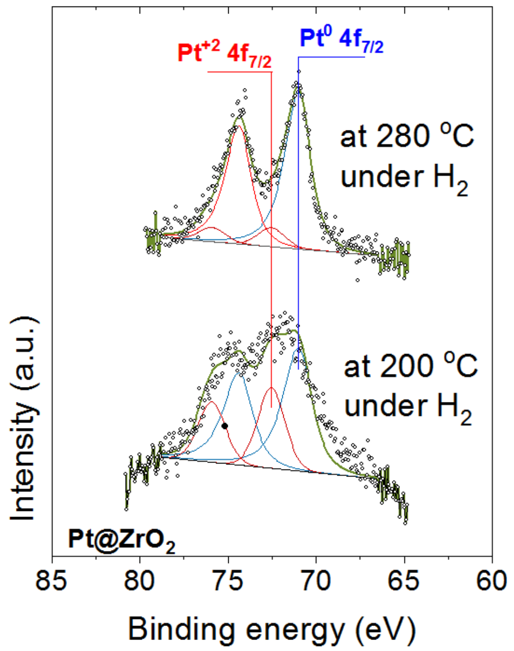

Figure S2. Pt 4f spectra of Pt@ZrO$_2$ at different temperatures and 1 mbar H_2.

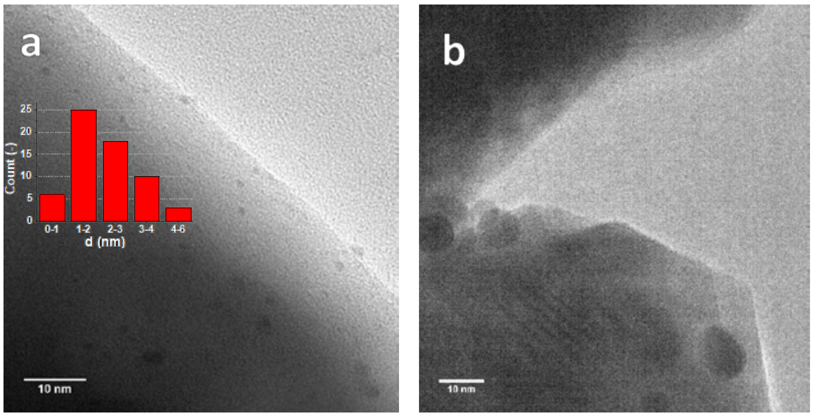

Figure S3. TEM images of (a) fresh and (b) used WI-PtNPs@ZrO$_2$ catalysts. (Inset of S2a: Pt NPs size distribution histogram).

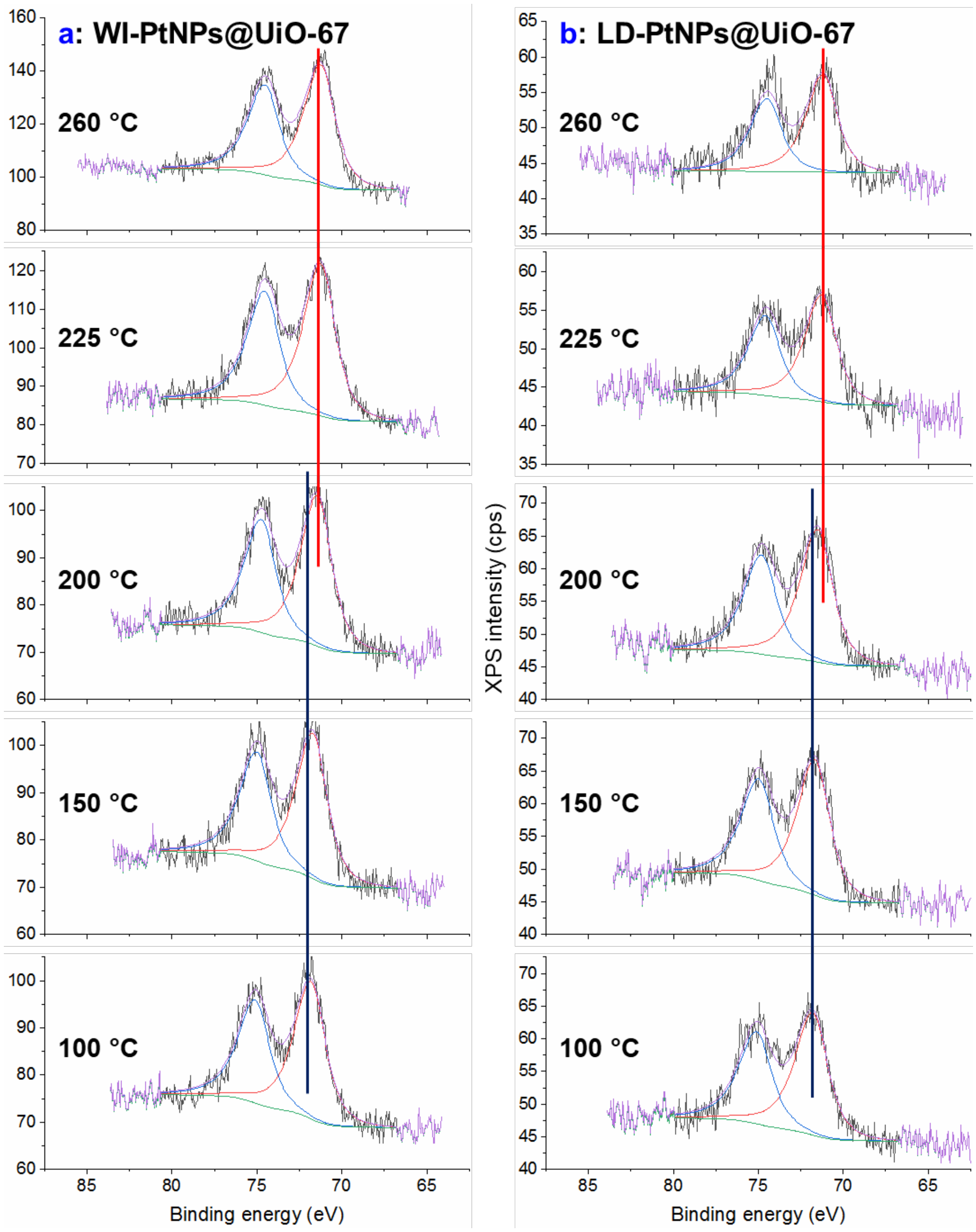

Figure S4. Pt 4f region for *operando* NAP-XPS temperature programmed CO oxidation over (a) WI-PtNPs@UiO-67 and (b) LD-PtNPs@UiO-67.

The geometry of NAP flow cell (Figure S5) is designed to maximise the collection of photoelectrons, and the gas and catalyst are not well mixed providing a bypass of the catalyst bed. Nevertheless, both experiments confirm the superior catalytic activity of LD-PtNPs@UiO-67 over WI-PtNPs@UiO-67.

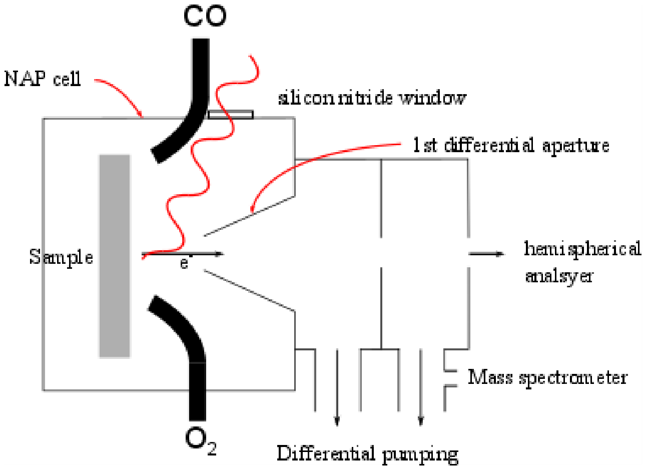

Figure S5. Schematic diagram of the configuration of the flow cell used in NAP-XPS measurements.

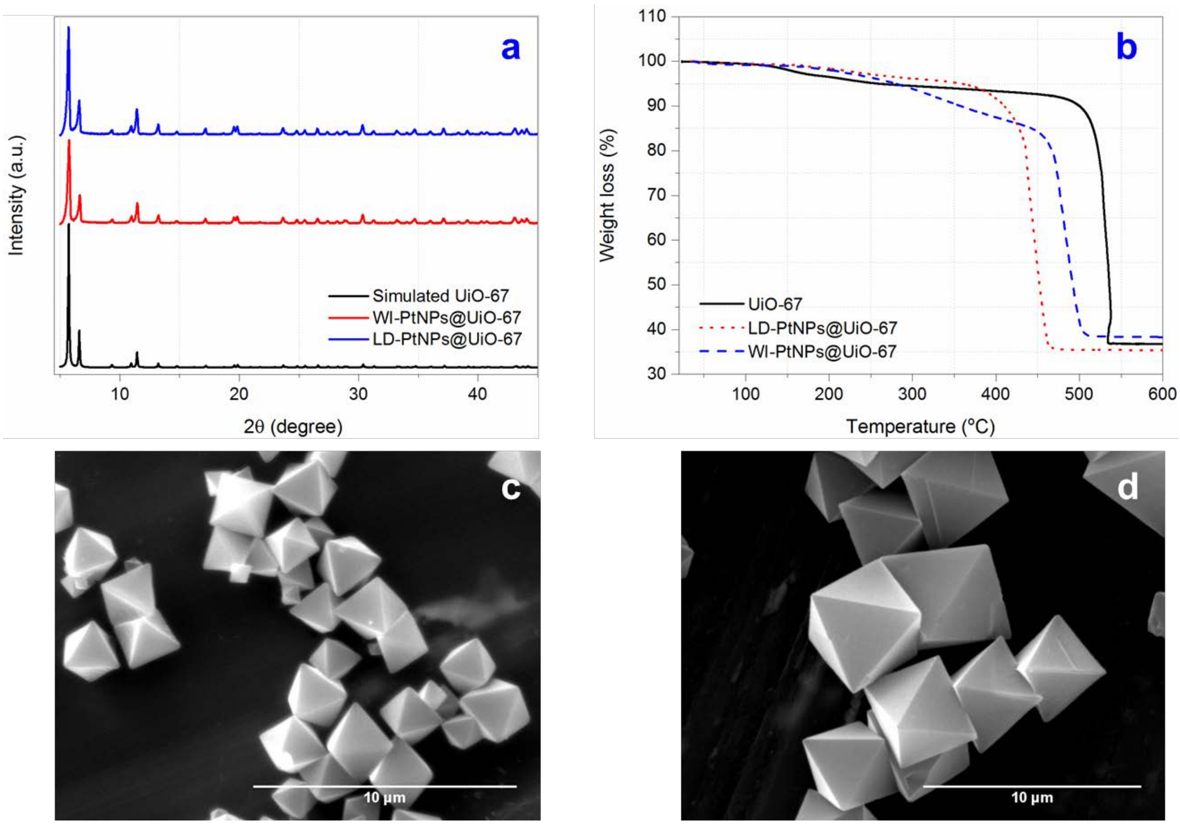

Figure S6. (a) XRD patterns of the simulated UiO-67 and UiO MOFs based catalysts, (b) TGA Curves of Pt@UiO-67 catalysts, SEM images of catalysts prepared by WI (c) and LD (d).

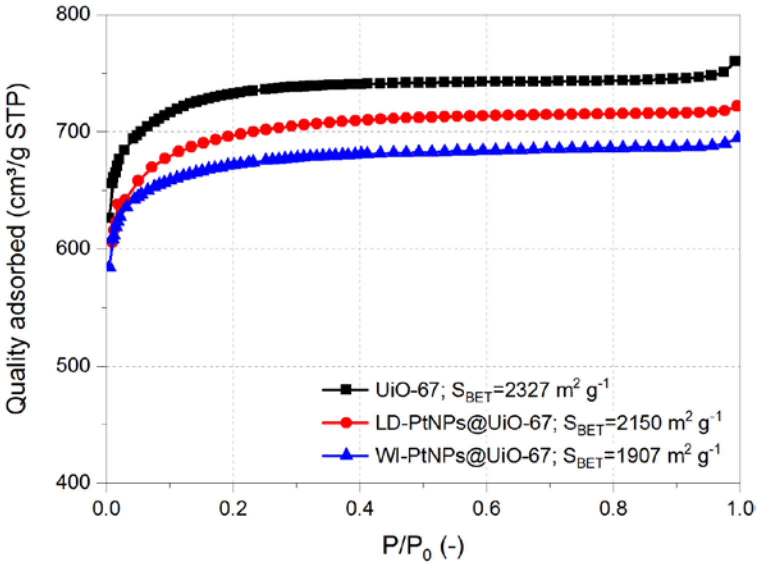

Figure S7. Nitrogen sorption isotherms of materials measured at −196.15 °C.

The effect of Pt precusors, catalysts preparation and reduction methods on the catalyst and acativty:

The method with $Pt(acac)_2$ and acetone was used (for the preparation of WI-Pt@UiO-67 catalyst) due to the wettability issue with the K_2PtCl_4 and water system (which was used for the ZrO_2 based catalyst) for impregnating UiO-67. In order to examine the effect of Pt precursors on the catalytic performance of wet impregnated UiO-67 catalysts, we prepared another reference WI-Pt@UiO-67catalyst using the method described below.

'UiO-67 support was synthesised by a microwave-assisted method proposed in our previous work. To prepare WI-Pt@UiO-67, 4.3 mg K_2PtCl_4 was dissolved in 2 ml DMF and then Pt precursor solution was dropped on 100 mg UiO-67 support stepwise. When the sample was fully moistened with the Pt precursor solution, it was dried in an oven at 120 °C before the next deposition. The WI-Pt@UiO-67 was reduced at 300 °C (heating ramp: 5 °C min^{-1}) under hydrogen-argon flow (flowrate: 70 ml min^{-1} (5% H_2), heating ramp: 5 °C min^{-1}) to give WI-PtNPs@UiO-67.'

In the light-off experiment, the two WI-Pt@UiO-67 catalysts using (based on $Pt(acac)_2$ (in acetone) and K_2PtCl_4 (in DMF) precursors) show comparable activity under the condition used, as shown in Figure S8.

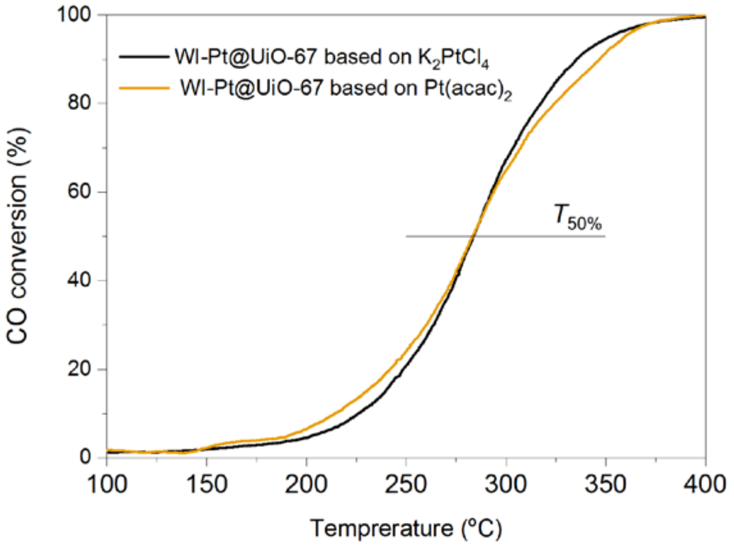

Figure S8. Comparison of light-off curves of CO oxidation over WI-Pt@UiO-67 catalysts prepared using different Pt precursors: Conditions: heating ramp = 6 °C min^{-1}, atmospheric pressure, total flowrate = 100 ml min^{-1}, CO/O_2 = 2, balanced using Ar.

For the thermal decomposition of platinum(II) acetylacetonate, 200 °C is sufficient to decompose acetylacetonates thermally into acetone, as previously reported in the literature.[5] Additionally, we also performed TG analysis of platinum(II) acetylacetonate, showing the sharp drop in weight loss at around 200 °C (Figure 2). Conversely, for the WI-PtNP@UiO-67 catalyst, there is no further weight loss at 200 °C (Figure 2). It is worth noting that the weight loss from 250 °C to 350 °C is related to dehydroxylation of UiO-67 and removal of monocarboxylate ligands,[1] instead of the weight loss related to the decomposition of Pt acetylacetonate precursor. Therefore, we can conclude that the pre-treatment procedure used in this study is capable of removing the acetylacetonate groups from the catalyst.

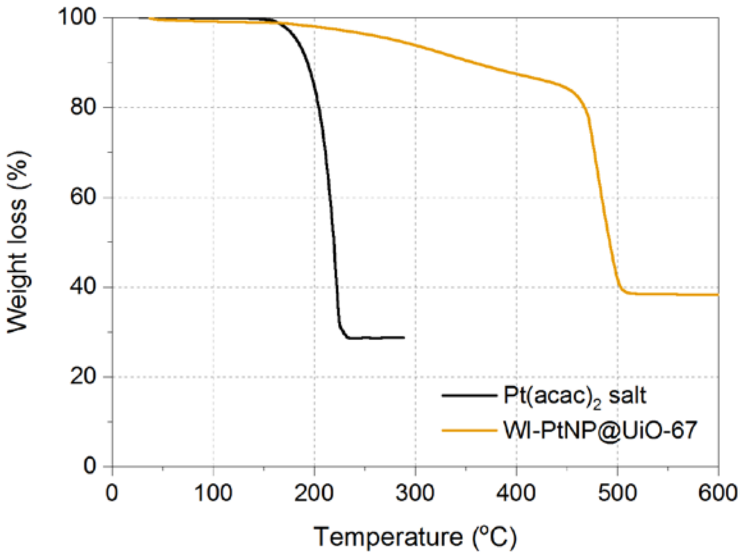

Figure S9. TGA curves of $Pt(acac)_2$ salt and the WI-PtNP@UiO-67 catalyst.

With regard to the Cl residues from Pt@ZrO_2 prepared using K_2PtCl_4, we performed detailed *ex situ* X-ray absorption fine structure (XAFS) spectroscopy of the as synthesised and reduced samples to show the effectiveness of the reduction step to remove Cl residues (Figures S11–12 and Table S1).

The XANES spectrum of PtNP@ZrO_2 (reduced catalyst) compares well with the Pt^0 foil reference. Fitting the EXAFS data of PtNP@ZrO_2 using 1st and 2nd shell Pt-Pt scattering paths and a multiple Pt-Pt-Pt scatter path provides a good fit to Pt metal. The coordination number of the 1st shell Pt-Pt path is 11, suggesting large metallic Pt NPs.

The as synthesised Pt/ZrO_2 fits well to 4 Cl paths at a distance of 2.3 Å, suggesting the Pt is still coordinated to the Cl's of the precursor, K_2PtCl_4. Looking at the XANES spectra, the white line intensity and features after the edge of Pt/ZrO_2 sample are very similar to that of K_2PtCl_4.

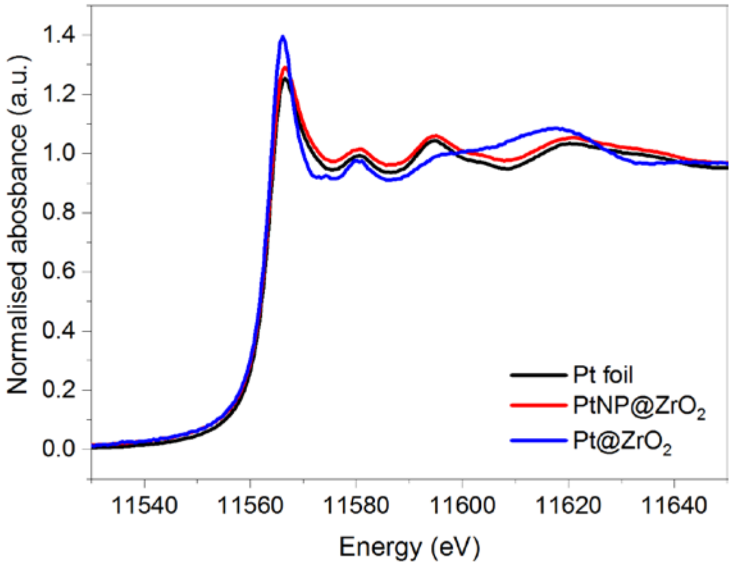

Figure S10. XANES spectra of Pt foil reference, Pt@ZrO_2 and PtNP@ZrO_2.

Table S1. EXAFS fit parameters.

Sample	Abso.-Scatt.	E_0	CN	R_{eff}	σ^2	R_{factor}
PtNP/ZrO_2 Fit X[a]	Pt-Pt1	7.6±0.5	11.2±0.5	2.762±0.002	0.0050±0.0002	0.011
	Pt-Pt2		6±2	3.90±0.01	0.007±0.002	
	Pt-Pt-Pt		18±5	4.810±0.009	0.008±0.001	
Pt/ZrO_2 Fit 20[b]	Pt_Cl	8.8±0.6	3.5±0.2	2.305±0.004	0.0019±0.0004	0.0098

[a] amplitude = 0.85, 3.5 <k <16.4, 1.15 < R < 4.3, number of independent points = 25.9

[b] amplitude = 0.85, 3.5< k <16.4, 1.15< R <3.0, number of independent points= 14.8

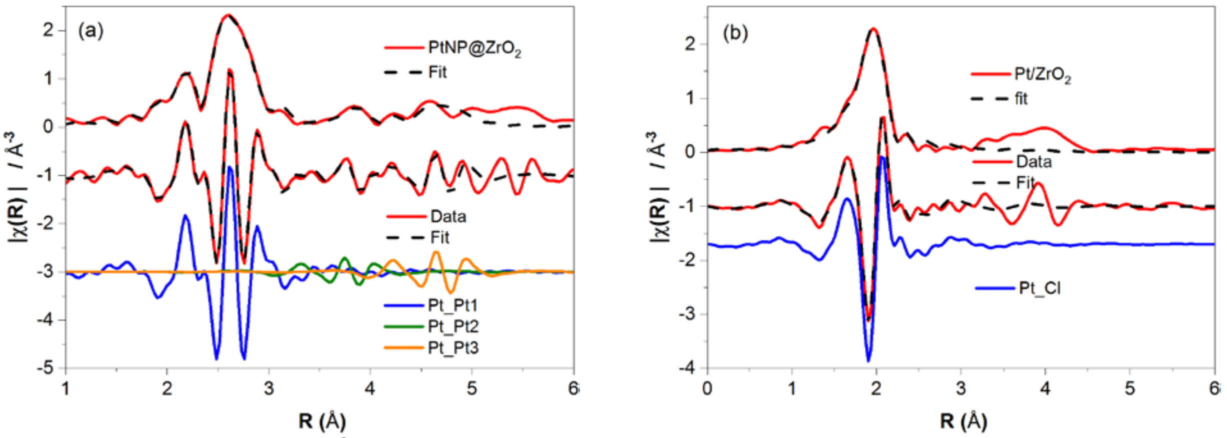

Figure S11. Magnitude of the k^2 weighted Fourier transform for the EXAFS data and fit for PtNP@ZrO_2 and Pt@ZrO_2. The imaginary part of the k^2 weighted FT of the data, fit and individual scattering paths is also shown for each.

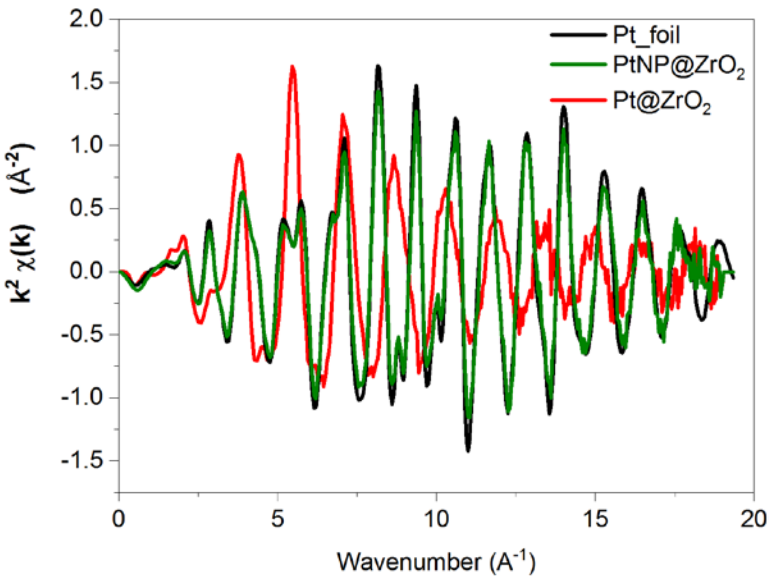

Figure S12. χ(k) data of Pt@ZrO_2, PtNP@$ZrO_2$and the Pt foil reference.

The χ(k) data of PtNP@ ZrO_2 and Pt@ ZrO_2 and the Pt foil are shown in Figure S12, showing that PtNP@ ZrO_2 resembles the Pt metal reference closely. Thus, we can conclude that the pre-treatment procedure used in this study can remove the chloride and acetylacetonate groups from the catalyst surface, addressing the concern raised by the reviewer. The SI was also updated with relevant information to explain the choice of method for catalyst preparation and the effect of Pt precursors and catalyst pre-treatment methods on the resulting catalysts and activity.

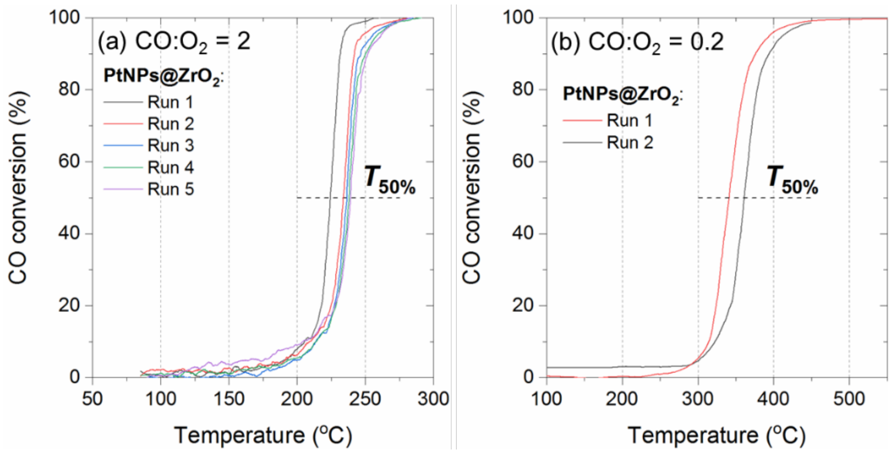

Figure S13. Light-off curves of CO oxidation over Pt catalysts under different oxidising conditions (a) stoichiometric and (b) lean condition.

References:

[1] R. Vakili, S. Xu, N. Al-Janabi, P. Gorgojo, S. M. Holmes, X. Fan, *Microporous and Mesoporous Materials* **2018**, *260*, 45-53.

[2] aS. Øien, G. Agostini, S. Svelle, E. Borfecchia, K. A. Lomachenko, L. Mino, E. Gallo, S. Bordiga, U. Olsbye, K. P. Lillerud, C. Lamberti, *Chemistry of Materials* **2015**, *27*, 1042–1056; bP. Hester, S. Xu, W. Liang, N. Al-Janabi, R. Vakili, P. Hill, C. A. Muryn, X. Chen, P. A. Martin, X. Fan, *Journal of Catalysis* **2016**, *340*, 85-94.

[3] C. H. Bartholomew, R. J. Farrauto, *Fundamentals of Industrial Catalytic Processes*, 2nd ed., John Wiley & Sons, Inc., Hoboken, New Jersey, USA, **2010**.

[4] L. Lukashuk, K. Föttinger, E. Kolar, C. Rameshan, D. Teschner, M. Hävecker, A. Knop-Gericke, N. Yigit, H. Li, E. McDermott, M. Stöger-Pollach, G. Rupprechter, *Journal of Catalysis* **2016**, *344*, 1-15.

[5] N. K. Sahu, A. Prakash, D. Bahadur, *Dalton Trans* **2014**, *43*, 4892-4900.
